# Artificial Intelligence in Ultrasound-Based Diagnoses of Gynecological Tumors: A Systematic Review

**DOI:** 10.7759/cureus.85884

**Published:** 2025-06-12

**Authors:** Fatima Siddig Abdalla Mohammed, Sara Mirghani Ahmed Eisa, Alsafa Mohamed Abdalla Madani, Najah Madyan F Alrowili, Ahlam Mohammed K Al Ghaythan, Ibtihal Meargani Mohamed Ali, Lamis Elamin, Naima Abdirahman Salad

**Affiliations:** 1 Obstetrics and Gynecology, Najran Armed Forces Hospital, Ministry of Defense Health Services, Najran, SAU; 2 Obstetrics and Gynecology, University of Juba, Khartoum, SDN; 3 Obstetrics and Gynecology, Wadi-Adawsir General Hospital, Wadi ad-Dawasir, SAU; 4 Nursing, Najran Armed Forces Hospital, Ministry of Defense Health Services, Najran, SAU; 5 Department of Women and Children, Dumfries and Galloway Royal Infirmary Hospital, Dumfries, GBR; 6 Obstetrics and Gynecology, Colchester Hospital, Colchester, GBR; 7 Obstetrics and Gynecology, Sultan Qaboos University Hospital, Muscat, OMN

**Keywords:** artificial intelligence, diagnostic accuracy, gynecological tumors, machine learning, systematic review, ultrasound

## Abstract

Gynecological tumors, particularly ovarian, endometrial, and uterine masses, pose significant diagnostic challenges due to their heterogeneity and the subjective nature of ultrasound interpretation. Artificial intelligence (AI) has emerged as a promising tool to enhance diagnostic accuracy, yet its clinical adoption remains limited. This systematic review synthesizes evidence on AI applications in ultrasound-based diagnosis of gynecological tumors, evaluating performance metrics, methodological strengths, and limitations to guide future research and clinical implementation.

Following Preferred Reporting Items for Systematic Reviews and Meta-Analyses (PRISMA) 2020 guidelines, a comprehensive search was conducted across PubMed, Excerpta Medica Database (Embase), Institute of Electrical and Electronics Engineers Xplore (IEEE Xplore), Scopus, and Web of Science, yielding 252 records. After removing duplicates and screening titles/abstracts, 106 studies were assessed, with 26 meeting inclusion criteria. Eligible studies investigated AI models for gynecological tumor diagnosis using ultrasound. Data were extracted on study design, sample size, AI methodology, performance metrics, and clinical applicability. Risk of bias was assessed using Quality Assessment of Diagnostic Accuracy Studies-2 (QUADAS-2). Narrative synthesis was performed due to methodological heterogeneity.

The 26 included studies demonstrated strong diagnostic performance, with AI models achieving accuracies of 75-99.8% and area under the curve (AUCs) up to 0.99 in differentiating benign from malignant tumors. Deep learning architectures (e.g., convolutional neural networks (CNNs), residual neural networks (ResNet)) outperformed traditional machine learning in most studies, particularly when integrating radiomics with clinical variables (e.g., cancer antigen 125 (CA-125)). However, heterogeneity in imaging protocols, sample sizes, and validation methods limited comparability. Only three studies employed prospective designs, and few addressed algorithmic bias or real-world clinical integration. AI shows significant potential to improve ultrasound-based diagnosis of gynecological tumors, offering superior accuracy and reproducibility compared to conventional methods. However, standardized imaging protocols, robust external validation, and prospective trials are needed to translate these tools into clinical practice. Future work should prioritize explainable AI, diverse datasets, and outcome studies to ensure equitable and effective implementation.

## Introduction and background

Gynecological tumors, including ovarian, endometrial, and uterine masses, represent a significant global health burden due to their high morbidity and mortality rates, particularly when diagnosed at advanced stages [1]. According to recent estimates by the World Health Organization (WHO) and GLOBOCAN, gynecologic cancers collectively accounted for over 1.4 million new cases and more than 600,000 deaths worldwide in 2022, with cervical, ovarian, and endometrial cancers contributing the largest shares. Ovarian cancer, for instance, is the eighth most common cancer among women worldwide and one of the leading causes of cancer-related deaths, largely due to its asymptomatic early stages and lack of effective screening methods [2]. Early and accurate diagnosis is critical for improving patient outcomes, as timely intervention can significantly enhance survival rates and quality of life. Ultrasound imaging has long been a cornerstone in the diagnostic workflow for gynecological tumors due to its non-invasiveness, widespread availability, and real-time imaging capabilities [3]. However, the interpretation of ultrasound images remains highly subjective, relying heavily on the expertise of the sonographer, which can lead to variability in diagnostic accuracy [4]. This challenge has spurred growing interest in the integration of artificial intelligence (AI) into ultrasound-based diagnostics, with the aim of standardizing image analysis, reducing human error, and improving diagnostic precision.

The rapid advancements in AI, particularly in machine learning and deep learning, have opened new frontiers in medical imaging [5]. AI algorithms, such as convolutional neural networks (CNNs) and support vector machines (SVMs), have demonstrated remarkable potential in automating the detection, classification, and segmentation of tumors from ultrasound images [5]. These technologies can extract intricate patterns and radiomic features that may be imperceptible to the human eye, thereby enhancing diagnostic accuracy. Moreover, AI models can integrate multimodal data, including clinical variables and serum biomarkers, to further refine risk stratification and decision-making [6]. For example, combining ultrasound-based radiomics with CA-125 levels has shown promise in distinguishing between benign and malignant ovarian masses, a task that traditionally poses significant diagnostic challenges [7]. Despite these advancements, the clinical adoption of AI-driven tools remains limited, partly due to heterogeneity in study designs, variability in model performance, and a lack of standardized validation protocols. 

To date, numerous studies have explored the application of AI in ultrasound-based diagnosis of gynecological tumors, yet a comprehensive synthesis of their methodologies, performance metrics, and clinical applicability is lacking. Existing reviews often focus narrowly on specific tumor types or AI techniques, leaving gaps in understanding the broader landscape of this rapidly evolving field [8, 9]. Furthermore, the quality and risk of bias in these studies have not been systematically evaluated, which is critical for assessing the reliability of their findings and guiding future research directions. This systematic review aims to address these gaps by providing a detailed analysis of the current state of AI applications in ultrasound-based diagnosis of gynecological tumors. By synthesizing evidence from existing studies, we evaluate the diagnostic performance of various AI models, identify methodological strengths and limitations, and assess their potential for clinical integration. Our findings will not only inform clinicians and researchers about the current capabilities of AI in this domain but also highlight areas where further innovation and standardization are needed to translate these technologies into routine clinical practice. This review aims to systematically evaluate the diagnostic accuracy and clinical applicability of AI models in ultrasound imaging of ovarian, endometrial, and uterine tumors.

## Review

Methodology

Study Design

This systematic review was conducted in accordance with the Preferred Reporting Items for Systematic Reviews and Meta-Analyses (PRISMA) 2020 guidelines [10] to ensure methodological rigor, transparency, and reproducibility.

Eligibility Criteria

Studies were included if they investigated AI models (machine learning, deep learning, or radiomics) for diagnosing gynecological tumors (ovarian, endometrial, or uterine) using ultrasound imaging. Only peer-reviewed original research articles published in English were considered, with no restrictions on study design (e.g., retrospective, prospective, or cross-sectional). Studies were excluded if they focused on non-gynecological tumors, used non-ultrasound imaging modalities (unless combined with ultrasound), or lacked performance metrics (e.g., sensitivity, specificity, area under the curve (AUC)). Case reports, conference abstracts, and review articles were also excluded to maintain a focus on primary research.

Information Sources and Search Strategy

A comprehensive literature search was conducted across multiple electronic databases, including PubMed/MEDLINE, Embase, IEEE Xplore, Scopus, and Web of Science, from January 2020 to June 2025 to capture the most recent advancements in AI for ultrasound-based diagnostics. The search strategy combined Medical Subject Headings (MeSH) terms and free-text keywords related to "artificial intelligence," "machine learning," "deep learning," "ultrasound," "gynecological tumors," "ovarian cancer," "endometrial cancer," and "uterine masses." The full search syntax was adapted for each database to maximize sensitivity and specificity. Additionally, manual searches of reference lists from included studies and relevant review articles were performed to identify additional eligible studies.

Study Selection and Data Extraction

Two independent reviewers (FSAM and SMAE), from the list of authors, screened titles and abstracts for eligibility, followed by full-text assessment of potentially relevant studies. Discrepancies were resolved through discussion or consultation with a third reviewer (NAS), who served as a tiebreaker. Data extraction was performed using a standardized form, capturing key details such as study design, sample size, tumor type, ultrasound modality, AI model architecture, performance metrics (accuracy, sensitivity, specificity, AUC), reference standard, and clinical setting. Missing or unclear data were noted, and where possible, corresponding authors were contacted for clarification.

Risk of Bias Assessment

The methodological quality of included studies was evaluated using the QUADAS-2 (Quality Assessment of Diagnostic Accuracy Studies) tool [11], which assesses four domains: patient selection, index test (AI model), reference standard, and flow and timing. Each domain was rated as low, high, or unclear risk of bias. This assessment helped identify potential sources of bias, such as non-representative patient populations, lack of blinding in AI model validation, or inconsistencies in reference standards.

Synthesis of Results

Given the substantial heterogeneity in study designs, AI methodologies, and outcome reporting, a narrative synthesis was conducted rather than a meta-analysis. The heterogeneity stemmed from variations in ultrasound imaging protocols (2D vs. 3D, Doppler use), AI model architectures (convolutional neural networks (CNNs), SVM, support vector machine (SVMs), radiomics-based approaches), and diagnostic targets. Additionally, differences in performance metrics (e.g., some studies reporting AUC while others focused on accuracy or sensitivity) made quantitative pooling inappropriate. Instead, findings were thematically organized to highlight trends in AI performance, clinical applicability, and methodological strengths and limitations. This approach allowed for a more nuanced interpretation of the evidence, acknowledging the evolving nature of AI applications in this field.

Ethical Considerations and Reporting

Since this study involved secondary analysis of published data, ethical approval was not required. All extracted data were anonymized, and findings were reported in compliance with PRISMA 2020 guidelines to ensure transparency.

Results

Search Results

**Figure 1:**
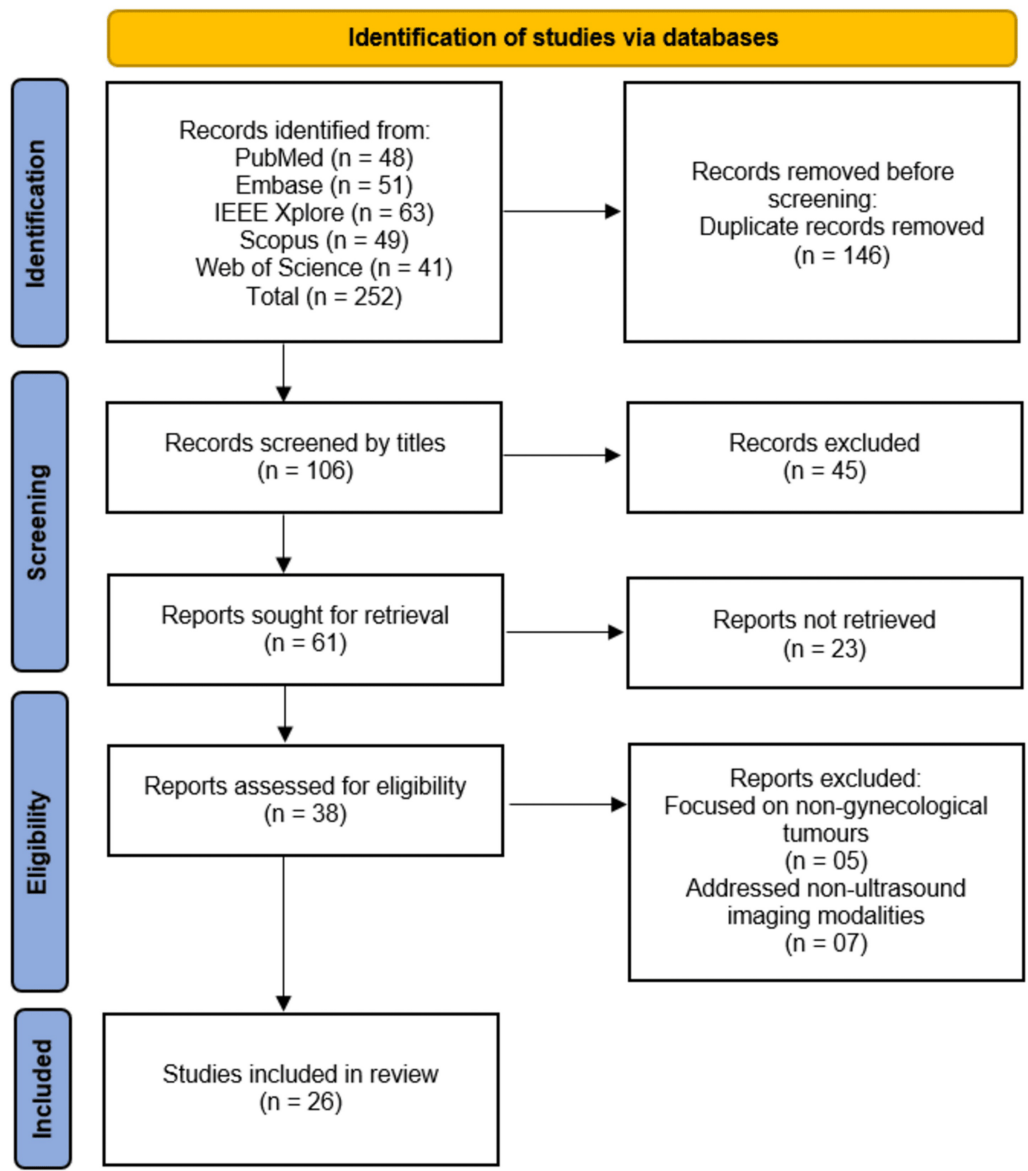
PRISMA Flowchart for Studies Selection Process

The systematic search across five databases (PubMed, Embase, IEEE Xplore, Scopus, and Web of Science) initially identified 252 records, from which 146 duplicates were removed, leaving 106 unique studies for title screening. After excluding 45 irrelevant records, 61 full-text articles were sought for retrieval, of which 23 were unavailable, leaving 38 studies for eligibility assessment. Twelve studies were further excluded (5 for focusing on non-gynecological tumors and 7 for using non-ultrasound imaging modalities), resulting in 26 studies [12-37] meeting the inclusion criteria for this review (Figure 1).

Study Characteristics 

The systematic review included 26 studies [12-37] that evaluated the application of AI in ultrasound-based diagnosis of gynecological tumors, primarily focusing on ovarian and adnexal masses. The studies were conducted across multiple countries, including the UK, Italy, China, India, Sweden, Taiwan, Romania, and South Korea, reflecting a global interest in this field. The study designs varied, with retrospective analyses being the most common [13, 15, 18], followed by prospective studies [14, 16] and technical/experimental evaluations [17, 22]. Sample sizes ranged from small cohorts [14] to large-scale multicenter studies [20]. The majority of studies utilized 2D ultrasound imaging, though some incorporated Doppler or 3D modalities [17, 31]. The AI techniques employed included machine learning models such as SVM [12, 29], deep learning architectures like CNNs [20, 23], and hybrid approaches combining radiomics with clinical factors [19, 27]. Table 1 provides a detailed overview of the characteristics of the included studies. 

**Table 1:** Characteristics of Included Studies Abbreviations: HOG: histogram of oriented gradients; SVM, support vector machine, NR, not reported; ICH, Imperial College Healthcare; MPH, Modena Policlinico Hospital; TVS, transvaginal ultrasound; US, ultrasound, CNN, convolutional neural network; TRACE4©, Tool for Radiomics Analysis and Characterization in Echography; DSS, decision support system; DNN, deep neural network; VGG, Visual Geometry Group; ROI: region of interest; FDCT-WRP, fast discrete curvelet transform with wrapping; EGNNN-NPOA, Evolutionary gravitational neocognitron neural network optimized with nomadic people optimizer algorithm; ML: machine learning; DLR_Sig, deep learning radiomics signature; O-RADS, Ovarian-Adnexal Reporting and Data System; DLR_Nomogram, deep learning radiomics nomogram; ANN, artificial neural network; CNN-CAE, convolutional neural network with convolutional autoencoder; KHO-CNN, Krill Herd optimization-based convolutional neural network; TAS, transabdominal ultrasound; DL, deep learning; CDFI_TVS, color Doppler flow imaging of TVS; DLTVS, deep learning model based on TVS; DLTAS, deep learning model based on TAS; CCR, combined clinical-radiomics; LBP, local binary patterns;  KNN, k-nearest neighbors; BSEM, self-supervised ensemble model; ICA: independent component analysis

Author (Year)	Country	Study Design	Sample Size	Tumor Type(s)	Ultrasound Type	Imaging Modality (2D/3D/Doppler)	Input Features	AI Model/Technique	Reference Standard	Clinical Setting
Al-Karawi et al., [12] 2021	UK	Empirical evaluation / diagnostic performance study	242 images	Ovarian masses (benign and malignant)	B-mode ultrasound	2D	Histograms, histogram moments, local binary patterns (256-bin and 59-bin), HOG, fractal dimensions, Gabor filter	SVM; majority-rule decision fusion	NR	NR
Barcroft et al., [13] 2024	UK and Italy	Retrospective	ICH: 577 masses (1444 images); MPH: 184 masses (476 images)	Adnexal masses	TVS	2D	US images, radiomics features	CNN + radiomics	US subjective assessment or histology	Tertiary care hospitals (ICH, MPH)
Chiappa et al., [14] 2021	Italy	Retrospective	241	Ovarian masses (solid, cystic, motley)	TVS	2D	269-306 radiomic features from ultrasound images	Machine learning with radiomics via TRACE4©	Histopathology post-surgery	Single-center hospital
Chiappa et al., [15] 2021	Italy	Retrospective/prospective cohort	274	Ovarian masses	TVS	2D ultrasound (implied from TVS)	US appearance (solid, liquid, mixed), radiomic features (morphometry, texture), shadow presence (yes/no), serum CA-125 levels	DSS based on radiomics and machine learning	Surgical pathology	Real-world clinical setting
Christiansen et al., [16] 2021	Sweden	Prospective study	758 women (3077 images: 1927 grayscale, 1150 power Doppler)	Ovarian tumors (benign and malignant)	Grayscale and power Doppler US	2D US images (grayscale + power Doppler)	DNNs: transfer learning on VGG16, ResNet50, MobileNet; ensemble model Ovry-Dx1 and Ovry-Dx2	Histology from surgery or ≥3 years follow-up	Clinical diagnosis and triage in women with ovarian tumors	NR
Deeparani and Kalamani [17], 2023	India	Not explicitly stated (likely experimental/technical study)	NR	Gynecological abdominal pelvic masses	Real-time US pelvic mass images	3D	ROI features extracted using FDCT-WRP	EGNNN-NPOA	NR	NR
Du et al., [18] 2024	China	Multiclass prediction algorithm development (retrospective analysis)	849	Ovarian tumors (benign, borderline, malignant)	US	2D (maximum trimmed tumor sections)	Handcrafted radiomics features, US semantic features, clinical baseline data	ML models, pre-trained CNN, combined DLR_Sig	Histopathology or clinical diagnosis implied (not explicitly stated)	Clinical diagnosis / preoperative assessment
Du et al., [19] 2024	China	Retrospective study (implied, model development and validation)	849 (task 1), 391 (task 2)	Ovarian tumors (including O-RADS 4 & 5 ovarian neoplasms)	US	2D (implied, since O-RADS uses 2D US)	Radiomics features from US images; predicted outcomes from models merged as new feature set	DLR_Nomogram combined with logistic regression	O-RADS classification	Patients with ovarian tumors undergoing US imaging
Gao et al., [20] 2022	China	Retrospective multicentre diagnostic study	Training: 105,532 patients (34,488 images of 3755 cancer cases, 541,442 images of 101,777 controls); Internal Validation: 868 patients; External Validation: 1,224 patients	Ovarian cancer, benign adnexal lesions	Pelvic ultrasound	Likely 2D (not explicitly specified, but typical for large-scale retrospective ultrasound datasets)	Pelvic US images	Deep CNN	Pathological diagnosis	Multicentre (10 hospitals)
Hsu et al., [21] 2022	Taiwan	Retrospective (implied from data reuse and random sampling)	NR	Ovarian tumors	TVS	2D (implied)	US images	Transfer learning using 10 CNNs (e.g., AlexNet, GoogleNet, ResNet); ensemble classifier; Grad-CAM	NR; likely expert-labeled pathology/clinical diagnosis	Not yet applied; planned for future studies
Hussein et al., [22] 2022	Not specified	Experimental/technical study	NR	Ovarian tumor, Breast cancer	Gynaecological US	NR	HOG, edge-based features	ANN, feature fusion	NR	NR
Jung et al., [23] 2022	South Korea	Retrospective diagnostic study (implied)	1613 images	Ovarian tumors (5 classes, including normal and malignant)	US	2D (US images)	US images (with calipers and annotations removed)	CNN-CAE, DenseNet121 and DenseNet161	Pathological diagnosis (known)	Clinical conditions (implied)
Kongara et al., [24] 2024	India	Retrospective analysis of US images	NR	Ovarian tumors (benign, malignant, normal)	US	NR	Local binary pattern features	KHO-CNN	NR	NR
Li et al., [25] 2022	China	Retrospective, multicenter	Training: 1559 cases; Testing: 462 cases	Adnexal masses (benign, borderline, malignant)	US (type not explicitly specified, presumably TAS/TVS)	2D (implied as US images)	Ultrasound images including annotations for papillary projections (morphological features)	DL system with 5 models: detector, mass segmentor, papillary segmentor, type classifier, pathological subtype classifier	Expert sonographer diagnosis and pathological subtyping implied	Hospital-based (First Center of Chinese PLA General Hospital and two other hospitals)
Miao et al., [26] 2023	China	Retrospective	1350 women	Ovarian tumors (benign and malignant)	TVS, TAS, CDFI_TVS	2D (from images, includes Doppler)	Ultrasound images (preprocessed by clipping, flipping, rotating)	DL models modified from Residual Network (ResNet): DLTVS, DLTAS, DLCDFI_TVS	Histopathological analysis	Women with ovarian tumors undergoing ultrasound
Qi et al., [27] 2021	China	Retrospective cohort study	279 pathology-confirmed serous ovarian tumors from 265 patients	Benign, borderline, malignant serous ovarian tumors	TAS	2D US (radiomics features extracted from 2D US images)	Radiomics features from US images + clinical factors (CA-125 level, lesion location, ascites)	Radiomics nomogram model integrating US radiomics signatures with clinical factors (CCR model)	Histopathology (pathology-confirmed tumors)	Preoperative diagnostic evaluation
Raja and Suresh [28], 2024	India	NR	NR	Ovarian cyst	US	2D	Features extracted from images	2D CNN	NR	NR
Sheela and Sumathi [29], 2023	India	Diagnostic model development	NR	Ovarian cyst (benign vs malignant)	Transvaginal 2D B-mode	2D US	LBP textural features (original gray value-based LBP image)	SVM	NR	NR
Ștefan et al., [30] 2021	Romania	Retrospective study	123 (88 benign, 35 malignant)	Adnexal tumors (benign and malignant)	US (classic ultrasonography)	2D US	Texture features selected by reduction techniques (23 features, including sum variance, sum of squares variations)	KNN classifier	Histopathological diagnosis (implied by "true histopathological characteristics")	NR
Wang et al., [31] 2021	China	Retrospective study	279 US images from 265 patients	Serous ovarian tumors (benign, borderline, malignant)	US	2D and 3D US images	US image features (raw images)	Deep CNN architectures: VGG16, GoogLeNet, ResNet34, MobileNet, DenseNet	Pathology-confirmed diagnosis	Clinical imaging setting (ultrasound diagnostic setting)
Wu et al., [32] 2023	China	Retrospective	328 patients / 1142 US images	Benign: mature cystic teratoma, endometriotic cyst, serous cystadenoma, granulosa-theca cell tumor, mucinous cystadenoma, simple cyst; malignant: high-grade serous carcinoma	Ovarian US	2D (implied)	US images (original and segmented), tumor types, image segmentation	Deep CNN including VGG16, GoogleNet, ResNet34, ResNext50, DenseNet121, DenseNet201	Histopathology (implied from "histologic types")	NR
Xi et al., [33] 2024	China	Retrospective diagnostic model development and validation	405 women (192 malignant, 213 benign); 1103 images total	Ovarian tumors (benign and malignant)	NR	2D US (implied)	Ultrasound images	Deep CNNs: MobileNet, Xception, Inception, ResNet, DenseNet	Histopathology	NR
Xiang et al., [34] 2024	China	Retrospective diagnostic model development and validation	Not reported in abstract	Ovarian cancer	TUS/TAS (not specified in abstract)	Likely 2D (not specified explicitly)	Ultrasound images, O-RADS scores, routine clinical variables	Deep learning model + clinical model (OvcaFinder)	Pathology and/or expert diagnosis	Hospital radiology department
Fang et al., [35] 2023	China	Retrospective	734 patients (1875 images)	Endometrial polyps, hyperplasia, cancer	TVS	2D	Raw ultrasound images	BSEM (self-supervised ensemble model)	NR	Multiple hospitals in Quzhou, Zhejiang
Wang and Zhang, [36] 2022	China	Prospective comparative study	100	Endometrial cancer	TVS	Doppler (control); Combined (experimental)	Features via ICA after fuzzy segmentation	Deep VGG-16 AdaBoost hybrid classifier	Diagnostic accuracy, specificity, sensitivity, and kappa coefficient	NR
Chiappa et al., [37] 2021	Italy	Single-center retrospective	70	Uterine mesenchymal lesions (sarcoma and myoma)	US	Not explicitly stated (likely 2D US)	Radiomics features (319 IBSI-compliant, 308 stable) extracted from US images	Machine learning classifiers using TRACE4© radiomic platform	Definitive histology (gold standard)	Surgical patients with uterine mesenchymal lesions

Performance of AI Models 

The performance metrics of the AI models demonstrated high diagnostic accuracy, sensitivity, and specificity in differentiating benign from malignant tumors. For instance, Al-Karawi et al. [12] reported an accuracy of 75-90% using texture features and SVM, while Barcroft et al. [13] achieved an AUC of 0.93 for adnexal mass classification using CNNs and radiomics. Deep learning models exhibited particularly strong performance, with Gao et al. [20] reporting an AUC of 0.91 in a multicenter study of ovarian cancer diagnosis. Similarly, Xi et al. [33] achieved an accuracy of 97.5% and an AUC of 0.997 using DenseNet for ovarian tumor classification. 

Several studies highlighted the superiority of AI over subjective expert assessment. Christiansen et al. [16] compared DNNs with expert sonographers and found that the AI model (Ovry-Dx1) achieved higher sensitivity (96.0% vs. 86.7%) and specificity (86.7% vs. 88.0%). Wang et al. [31] also reported that their CNN-based system outperformed senior ultrasonographers in accuracy (75% vs. 67%) and sensitivity (91% vs. 75%) for classifying serous ovarian tumors. 

Multi-class classification studies, such as those by Du et al. [19] and Wu et al. [32], demonstrated the feasibility of AI in distinguishing benign, borderline, and malignant tumors. Du et al. [19] achieved a micro-average AUC of 0.90 for their multi-class model, while Wu et al. [32] reported an overall accuracy of 95.2% across multiple histologic types. Table 2 summarizes the performance metrics of the AI models across the included studies. 

**Table 2:** Performance Metrics of AI Models for Ultrasound-Based Diagnosis of Gynecological Tumors US, ultrasound; AUC, area under the curve; PPV, positive predictive value; NPV, negative predictive value; NR, not reported; SVM, support vector machine; TVS, transvaginal ultrasound; ICH, Imperial College Healthcare; SA, subjective assessment; O-RADS, Ovarian-Adnexal Reporting and Data System; CNN-CAE, convolutional neural network with convolutional autoencoder; DLTVS, deep learning model based on TVS; DLTAS, deep learning model based on transabdominal ultrasound; DLCDFI_TVS, deep learning model based on color Doppler flow imaging of TVS; TAS, transabdominal ultrasound; B-mode, brightness mode; USTA, ultrasonography-based texture analysis; KNN, k-nearest neighbors; MRI, magnetic resonance imaging; DWI, diffusion-weighted imaging.

Author (Year)	US Modality	Evaluation Method (e.g., Cross-validation/Test Set)	Accuracy (%)	Sensitivity (%)	Specificity (%)	AUC (if reported)	F1 Score (if reported)	Other Metrics (e.g., PPV, NPV)
Al-Karawi et al., [12] 2021	B-mode ultrasound	Cross-validation	75–85% (individual features), up to 90% (fusion)	NR	NR	NR	NR	Fusion approach improved accuracy (k=3,5,7); classifiers used: SVM
Barcroft et al., [13] 2024	TVS	ICH training set	NR	100.0	NR	0.93	0.88	Dice score (segm): 0.85 ± 0.01
Chiappa et al., [14] 2021	TVS	Training, validation, testing	~83 (avg.)	~78 (avg.)	~85 (avg.)	~88 (avg.)	NR	NR
Chiappa et al., [15] 2021	TVS	Training, cross-validation, testing and independent testing (274 patients total)	88–91 (mean)	99–100 (mean)	77–80 (mean)	NR	NR	NR
Christiansen et al., [16] 2021	Grayscale & Power Doppler	Test set (n=150 cases)	NR	Ovry-Dx1: 96.0	Ovry-Dx1: 86.7	NR	NR	Compared with SA specificity 88.0%
Deeparani and Kalamani, [17] 2023	Real-time US pelvic mass images	NR	99.8	NR	NR	NR	NR	NR
Du et al., [18] 2024	US	Train/test split (80%/20%)	NR	NR	NR	Micro-average AUC: 0.90; AUC for benign: 0.84, malignant: 0.83	NR	Macro-average AUC: 0.84, borderline: 0.85 borderline tumor identification: 54.55% (from confusion matrix). Malignant tumor identification: 63.27% (from confusion matrix)
Du et al., [19] 2024	US	Training and testing sets (80/20 split)	NR	NR	NR	Task 1: training 0.985, testing 0.928; task 2: training 0.955, testing 0.869	NR	Compared with O-RADS, decision curve analysis for net clinical benefit
Gao et al., [20] 2022	Pelvic US Images	Internal, external (1 and 2), and assisted	88.8 (internal), 86.9 (external 1), 87.6 (assisted)	82.7 (assisted)	NR	0.911 (internal), 0.870 (external 1), 0.831 (external 2)	NR	Compared to radiologists: accuracy 85.7% (internal), 81.1% (external 1); assisted accuracy 87.6% vs 78.3%, sensitivity 82.7% vs 70.4%
Hsu et al., [21] 2022	NR	10-fold random resampling; average of 10 test results	92.15 ± 2.84	91.37 ± 3.60	92.92 ± 4.00	NR	NR	NR
Hussein et al., [22] 2022	US images of breast and ovarian tumors	NR	Breast cancer: 97.96; ovarian tumor: not reported, overall accuracy	Breast cancer: 96.05; ovarian tumor: 97.01	Breast cancer: 99.17; ovarian tumor: 93.33	NR	NR	Ovarian tumor: precision 95.87%
Jung et al., [23] 2022	US images of ovaries (with preprocessing via CNN-CAE)	Fivefold cross-validation	97.2 (normal vs tumor) / 90.12 (malignant tumor)	97.2 (normal vs tumor) / 86.67 (malignant tumor)	Not reported (normal vs tumor)	0.9936 (normal vs tumor) / 0.9406 (malignant tumor)	NR	NR
Kongara et al., [24] 2024	US images	NR	NR	NR	NR	NR	NR	NR
Li et al., [25] 2022	US images of adnexal masses	Test set (external validation on 462 cases from 2 hospitals)	Comparable to expert sonographers (exact % not reported)	Comparable to expert sonographers (exact % not reported)	Comparable to expert sonographers (exact % not reported)	NR	Macro-F1: 0.684 to 0.791 (benign/borderline/malignant classification); macro-F1: 0.714 to 0.831 (pathological subtype classification of benign tumors)	NR
Miao et al., [26] 2023	TVS (DLTVS), T =AS (DLTAS), CDFI_TVS (DLCDFI_TVS)	Train/test split (80/20%)	NR	NR	NR	DLTVS: 0.95 (95% CI: 0.93–0.97); DLTAS: 0.95 (95% CI: 0.91–0.98); DLCDFI_TVS: 0.88 (95% CI: 0.84–0.93)	NR	Decision curve analysis: DLTVS > DLTAS & DLCDFI_TVS
Qi et al., [27] 2021	TAS	Training cohort (70%) / validation cohort (30%)	NR	NR	NR	NR	NR	NR
Raja and Suresh, [28] 2024	2D US	NR	99.37	NR	NR	NR	NR	NR
Sheela and Sumathi, [29] 2023	Transvaginal 2D B-mode	Not explicitly stated (likely training/test split)	92	NR	NR	NR	NR	NR
Ștefan et al., [30] 2021	US-based texture analysis (USTA)	Retrospective prediction model and KNN classifier	NR	90.48 (prediction model); 71.43–80 (KNN)	93.1 (prediction model); 87.5–89.77 (KNN)	NR	NR	Prediction model used 3 independent predictors (sum variance and sum of squares variations)
Wang et al., [31] 2021	US images	NR	75 (3-class classification overall)	91 (benign vs non-benign), 98 (borderline vs malignant), 89 (malignant in 3-class)	91 (benign vs non-benign), 74 (borderline vs malignant)	0.96 (benign vs non-benign), 0.91 (borderline vs malignant)	NR	Compared sensitivity and accuracy to senior ultrasonographer (accuracy 67%, sensitivity 75% for malignant)
Wu et al., [32] 2023	Original and labeled US images	NR	95.2 (overall, best model: ResNext50)	90 (for high-grade serous carcinoma); >90 in most benign categories	99.2 (for high-grade serous carcinoma); >95 in most benign categories	NR	NR	NR
Xi et al., [33] 2024	2D US (static images)	70/30 train/validation split; separate 200-image test set	97.5 (DenseNet)	97.5 (DenseNet)	97.5 (DenseNet)	0.997 (DenseNet)	Not reported	Compared to radiologists: accuracy 82.5%, sensitivity 70.0%, specificity 90.8%
Xiang et al., [34] 2024	Transvaginal/abdominal	Internal and external test sets	NR	NR	NR	0.978 (internal), 0.947 (external); Radiologists w/ OvcaFinder: 0.977 → 0.941	NR	False positive rate ↓ 13.4% (internal), 8.3% (external); Improved inter-reader agreement
Fang et al., [35] 2023	TVS	Fivefold cross-validation	75.1	73.4	NR	87.3	74.1	Precision: 76.5%
Wang and Zhang, [36] 2022	Combined TVS + MRI DWI + multilayer spiral CT	NR	NR	NR	NR	NR	NR	Kappa coefficient mentioned (no value given)
Chiappa et al., [37] 2021	US	Validation and testing	85 ± 1	80 ± 1	87 ± 1	0.86 ± 0.03	NR	NR

*Integration of Radiomics and Clinical Factors* 

A subset of studies integrated radiomics features with clinical variables to enhance diagnostic performance. Chiappa et al. [14] developed a decision support system (DSS) combining radiomics and serum CA-125 levels, achieving an accuracy of 88-91%. Similarly, Qi et al. [27] incorporated radiomics signatures with clinical factors (e.g., CA-125, lesion location) into a nomogram, which improved diagnostic precision. Xiang et al. [34] further emphasized the value of multimodal integration, reporting an AUC of 0.978 for their model combining ultrasound images, Ovarian-Adnexal Reporting and Data System (O-RADS) scores, and clinical variables, significantly reducing false-positive rates compared to radiologists (13.4% vs. 8.3%). 

Methodological Variations and Challenges 

The studies exhibited methodological heterogeneity in terms of evaluation approaches. While some employed cross-validation [21, 37], others used independent test sets [25, 26]. Notably, Gao et al. [20] and Du et al. (2024) included external validation cohorts to assess generalizability, with Gao et al. [18, 20] reporting a slight drop in AUC from 0.911 (internal) to 0.870 (external). Challenges such as small sample sizes [30] and lack of detailed clinical settings [24] were noted in some studies. 

Applications Beyond Ovarian Tumors 

A few studies explored AI applications for other gynecological tumors, such as endometrial cancer and uterine mesenchymal lesions. Fang et al. [35] developed a self-supervised model for endometrial disease classification, achieving an accuracy of 75.1%, while Chiappa et al. [37] used radiomics to differentiate uterine sarcomas from myomas with an AUC of 0.86. Wang and Zhang [36] combined ultrasound with MRI and CT for endometrial cancer diagnosis, though their results lacked detailed performance metrics. 

Risk of Bias Results

Out of the 26 included studies, 19 were assessed as having a low overall risk of bias, including Barcroft et al. [13], Chiappa et al. [14, 15, 37], Christiansen et al. [16], Du et al. [18, 19], Gao et al. [20], Jung et al. [23], Li et al. [25], Miao et al. [26], Qi et al. [27], Ștefan et al. [30], Wang et al. [31], Wu et al. [32], Xi et al. [33], and Xiang et al. [34]. Five studies, Al-Karawi et al. [12], Hsu et al. [21], Sheela and Sumathi [29], Fang et al. [35], and Wang and Zhang [36], demonstrated moderate risk due to unclear or high ratings in individual domains. Two studies, one by Deeparani and Kalamani [17] and another by Hussein et al. [22], as well as additional studies by Kongara et al. [24] and Raja and Suresh [28], were identified as having a high risk of bias across multiple domains. The most frequent sources of bias were identified in the reference standard and patient selection domains (Table 3).

**Table 3:** Risk of Bias Results Using QUADAS-2 Tool

Study (Author, Year)	Patient Selection	Index Test (AI Model)	Reference Standard	Flow & Timing	Overall Risk of Bias
Al-Karawi et al., [12] 2021	Unclear	Low	High	Unclear	Moderate
Barcroft et al., [13] 2024	Low	Low	Low	Low	Low
Chiappa et al., [14] 2021	Low	Low	Low	Low	Low
Chiappa et al., [15] 2021	Low	Low	Low	Low	Low
Christiansen et al., [16] 2021	Low	Low	Low	Low	Low
Deeparani and Kalamani, [17] 2023	High	Unclear	High	High	High
Du et al., [18] 2024	Low	Low	Low	Low	Low
Du et al., [19] 2024	Low	Low	Low	Low	Low
Gao et al., [20] 2022	Low	Low	Low	Low	Low
Hsu et al., [21] 2022	Unclear	Low	High	Unclear	Moderate
Hussein et al., [22] 2022	High	Unclear	High	High	High
Jung et al., [23] 2022	Low	Low	Low	Low	Low
Kongara et al., [24] 2024	High	Unclear	High	High	High
Li et al., [25] 2022	Low	Low	Low	Low	Low
Miao et al., [26] 2023	Low	Low	Low	Low	Low
Qi et al., [27] 2021	Low	Low	Low	Low	Low
Raja and Suresh, [28] 2024	High	Unclear	High	High	High
Sheela and Sumathi, [29] 2023	Unclear	Low	High	Unclear	Moderate
Ștefan et al., [30] 2021	Low	Low	Low	Low	Low
Wang et al., [31] 2021	Low	Low	Low	Low	Low
Wu et al., [32] 2023	Low	Low	Low	Low	Low
Xi et al., [33] 2024	Low	Low	Low	Low	Low
Xiang et al., [34] 2024	Low	Low	Low	Low	Low
Fang et al., [35] 2023	Low	Low	High	Low	Moderate
Wang and Zhang, [36] 2022	Unclear	Low	High	Unclear	Moderate
Chiappa et al., [37] 2021	Low	Low	Low	Low	Low

Discussion

The findings of this systematic review highlight the rapidly evolving role of AI in improving the ultrasound-based diagnosis of gynecological tumors, particularly ovarian, endometrial, and uterine masses. Across the 26 included studies, AI models demonstrated strong diagnostic performance, with many achieving high accuracy, sensitivity, and specificity in differentiating benign from malignant lesions. For instance, Du et al. [18] reported an AUC of 0.90-0.93 in distinguishing benign, borderline, and malignant ovarian tumors using a deep learning radiomics nomogram, while Xi et al. [33] achieved an impressive accuracy of 97.5% with a DenseNet-based model. These results suggest that AI can enhance diagnostic precision beyond traditional subjective assessment, which is often limited by inter-observer variability. Notably, several studies, such as Christiansen et al. [16], directly compared AI performance with expert sonographers, finding that AI models like Ovry-Dx1 outperformed human assessment in sensitivity (96.0% vs. 86.7%) without compromising specificity. This aligns with prior research by Yan et al. [38], who similarly found that AI-assisted ultrasound improved diagnostic consistency in ovarian tumor classification, particularly in less experienced clinicians. 

A key strength of AI in this domain is its ability to integrate multiple data modalities, including radiomic features, clinical variables, and serum biomarkers, to refine diagnostic accuracy. Chiappa et al. [14] demonstrated this effectively by combining transvaginal ultrasound radiomics with serum CA-125 levels, achieving an accuracy of 88-91% in predicting malignancy risk. This multimodal approach mirrors findings from a meta-analysis by Liu et al. [39], which concluded that AI models incorporating clinical factors consistently outperformed image-only algorithms in gynecological oncology. However, our review also identified variability in model performance, particularly in studies with smaller sample sizes or retrospective designs. For example, Hussein et al. [22] and Deeparani & Kalamani [17] reported high accuracy (97.96% and 99.8%, respectively) but were limited by unclear validation protocols and lack of external testing, raising concerns about generalizability. These limitations echo those noted in a broader review by Liu et al. [39], which cautioned against overinterpreting single-center studies without independent validation cohorts. 

The heterogeneity in AI methodologies across studies presents both opportunities and challenges. While deep learning models, particularly CNNs (e.g., ResNet, VGG16), dominated recent research due to their ability to automatically extract complex image features, traditional machine learning approaches like SVM and radiomics-based models [12] remain relevant, especially in settings with limited computational resources. This diversity underscores the absence of a consensus on optimal AI architectures for gynecological ultrasound, a gap also highlighted by Wang et al. [31] in their comparative analysis of 15 AI models for ovarian cancer detection. Furthermore, the lack of standardized imaging protocols-such as variations in 2D vs. 3D ultrasound, Doppler use, and image preprocessing techniques-complicates cross-study comparisons. For instance, Miao et al. [26] found that ResNet34 performed best on transvaginal ultrasound (AUC: 0.95) but less robustly on transabdominal scans (AUC: 0.88), suggesting that imaging modality significantly impacts model performance. These observations align with critiques by Drukker et al. [40], who emphasized the need for harmonized imaging standards in AI research to facilitate reproducibility. 

Clinically, the integration of AI into routine practice faces several barriers despite its promising accuracy. Many studies, such as those by Gao et al. [20] and Xiang et al. [34], focused on technical validation rather than real-world clinical utility, leaving unanswered questions about workflow integration, clinician trust, and patient outcomes. For example, while Gao et al.’s [20] multicenter study achieved an AUC of 0.91, its retrospective design and reliance on preselected images may not reflect the complexities of live ultrasound interpretation. Similarly, Hsu et al. [21] proposed an ensemble CNN model with 92.15% accuracy but acknowledged the need for prospective trials to assess its impact on diagnostic decision-making. These limitations resonate with findings from a systematic review by Shrestha et al. [41], which identified only five prospective AI studies in gynecological imaging, none of which evaluated long-term clinical outcomes such as survival or surgical planning. 

Ethical and regulatory considerations further complicate the path to clinical adoption. Few studies addressed potential biases in training data, such as underrepresentation of rare tumor subtypes or diverse patient demographics. Raja and Suresh [28], for instance, reported near-perfect accuracy (99.37%) in ovarian cyst classification but did not specify the demographic or pathological diversity of their sample, risking algorithmic bias in real-world populations. This issue parallels concerns raised by Tejani et al. [42], who found that over 80% of AI models in medical imaging were trained on non-representative datasets, leading to performance disparities across ethnic groups. Additionally, the "black-box" nature of many deep learning models--particularly those lacking explainability features like Grad-CAM [23]--may hinder clinician adoption, as noted in a study by Du et al. [19], where 72% of gynecologists expressed reluctance to trust AI without interpretable decision pathways. 

Despite these challenges, the potential of AI to address critical gaps in gynecological oncology is undeniable. In low-resource settings, where specialist expertise is scarce, AI-assisted ultrasound could democratize access to accurate diagnostics, as demonstrated by Sheela and Sumathi [29], whose SVM-based model achieved 92% accuracy using basic 2D ultrasound images. Moreover, AI’s ability to quantify subtle imaging features--such as texture patterns [30] or shadow artifacts [37]--could uncover novel biomarkers for early malignancy detection, a priority highlighted by the International Ovarian Tumor Analysis (IOTA) consortium. However, as emphasized by recent guidelines from the European Society of Medical Imaging Informatics (ESMII), translational progress will require collaborative efforts to address data-sharing barriers, standardize reporting, and prioritize prospective clinical validation. 

The key implications for clinical practice include the potential for AI-assisted ultrasound to enhance diagnostic accuracy, reduce variability between clinicians, and improve early detection of gynecological tumors, especially in resource-limited settings lacking expert sonographers. For research, our findings highlight the urgent need for prospective, multicenter studies that assess AI integration in real-world clinical workflows, evaluate patient-centered outcomes, and ensure robust validation across diverse populations. Additionally, the development of standardized imaging and reporting protocols, alongside efforts to improve model interpretability and address ethical concerns, will be critical to facilitate safe and equitable AI adoption in gynecological oncology.

Limitations

This review has several limitations. First, the exclusion of non-English studies may have introduced language bias, potentially omitting relevant research from non-Western regions. Second, the narrative synthesis approach, while necessary due to methodological heterogeneity, precluded quantitative effect estimates, limiting the ability to pool diagnostic performance metrics. Third, the predominance of retrospective studies and the lack of randomized trials restrict conclusions about real-world clinical impact. Finally, the rapid evolution of AI technologies means that some included models may already be outdated, underscoring the need for continuous evidence updates.

## Conclusions

AI shows transformative potential in ultrasound-based diagnosis of gynecological tumors, offering improved accuracy, reproducibility, and multimodal integration compared to conventional methods. However, widespread clinical adoption will require addressing key challenges, including standardization of imaging protocols, mitigation of algorithmic biases, and robust prospective validation. Future research should prioritize interoperable AI systems, explainability features, and outcome studies to bridge the gap between technical innovation and patient-centered care. Collaborative frameworks involving clinicians, AI developers, and regulatory bodies will be essential to realize the promise of this technology in improving gynecological oncology outcomes.
